# Expansion of Natural Killer Cells in Peripheral Blood in a Japanese Elderly with Human T-Cell Lymphotropic Virus Type 1-Related Skin Lesions

**DOI:** 10.1155/2014/937513

**Published:** 2014-11-09

**Authors:** Shinsaku Imashuku, Naoko Kudo, Kagekatsu Kubo, Kouichi Ohshima

**Affiliations:** ^1^Division of Hematology, Takasago Seibu Hospital, 1-10-41 Nakasuji, Takasago 676-0812, Japan; ^2^Division of Internal Medicine, Takasago Seibu Hospital, Takasago 676-0812, Japan; ^3^Department of Pathology, School of Medicine, Kurume University, Kurume 830-0011, Japan

## Abstract

Natural killer (NK) cells were proposed to play an important role in the pathogenesis of human T-cell lymphotropic virus type 1- (HTLV-1-) associated neurologic disease. Our patient was a 77-year-old Japanese man, who had been treated for infective dermatitis associated with HTLV-1 for nearly 10 years. When referred to us, he had facial eczema/edema as well as extensive dermatitis at the neck/upper chest and nuchal area/upper back regions. Dermal lesions had CD3+CD4+ cells, but no NK cells. Flow cytometry of his peripheral blood showed a phenotype of CD2+ (97%), CD3+ (17%), CD4+ (12%), CD7+ (94%), CD8+ (6%), CD11c+ (70%), CD16+ (82%), CD19+ (0%), CD20+ (0%), CD56+ (67%), HLA-DR+ (68%), and NKp46+ (36%). Absolute numbers of CD56+NK cells in the peripheral blood were in a range of 986/*μ*L–1,270/*μ*L. The expanded NK cells in the peripheral blood are considered to be reactive, to maintain the confinement of the HTLV-1-positive CD4+ cells in the skin, and to prevent the progression of the disease.

## 1. Introduction

Among human T-cell lymphotropic virus type 1 (HTLV-1) infected individuals, adult T-cell leukemia/lymphoma (ATLL), and a chronic neurological disease, the HTLV-1-associated myelopathy/tropical spastic paraparesis (HAM/TSP) are two major symptomatic disorders. In addition, dermatological disorders are noted in 5–10% of the HTLV-1 infected patients [1]. The skin lesions could be either infectious or autoimmune dermatitis [2]. The infective dermatitis associated with HTLV-1 (IDH) is a chronic recurrent form of eczema affecting the scalp and retroauricular regions [1]. IDH may also progress to HAM/TSP or to ATLL [3]. Immunological features of IDH are similar to those of HAM/TSP [2]. However, expansion of natural killer (NK) cells has rarely been described in patients with IDH.

## 2. Case Report

**Figure 1:**
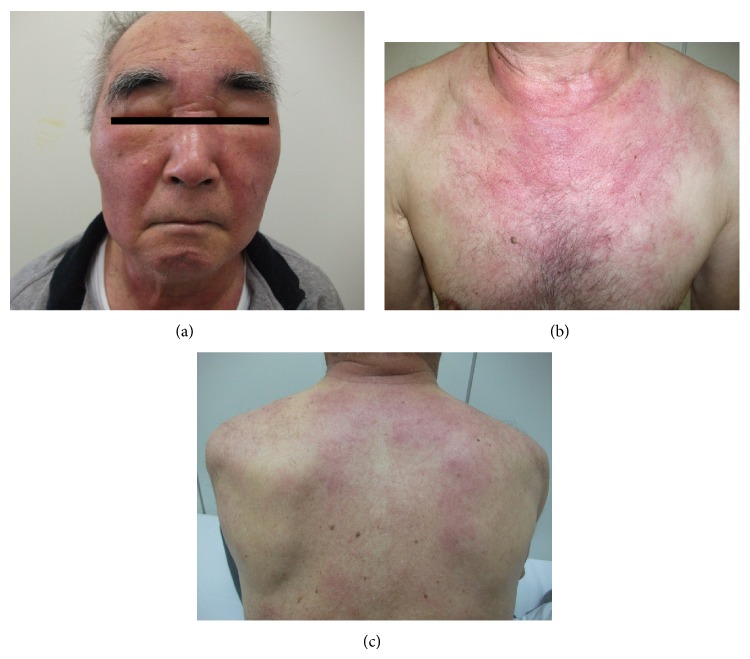
Facial edema/rash (a) and skin lesions at the neck/upper chest (b) as well as at the upper back (c).

**Figure 2:**
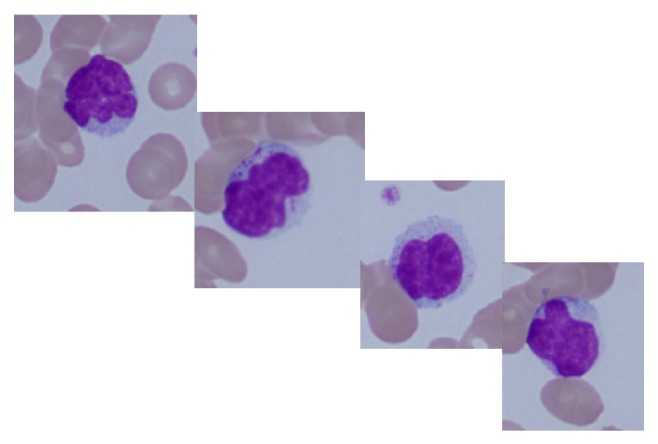
NK cells with azurophilic granules in the peripheral blood.

**Figure 3:**
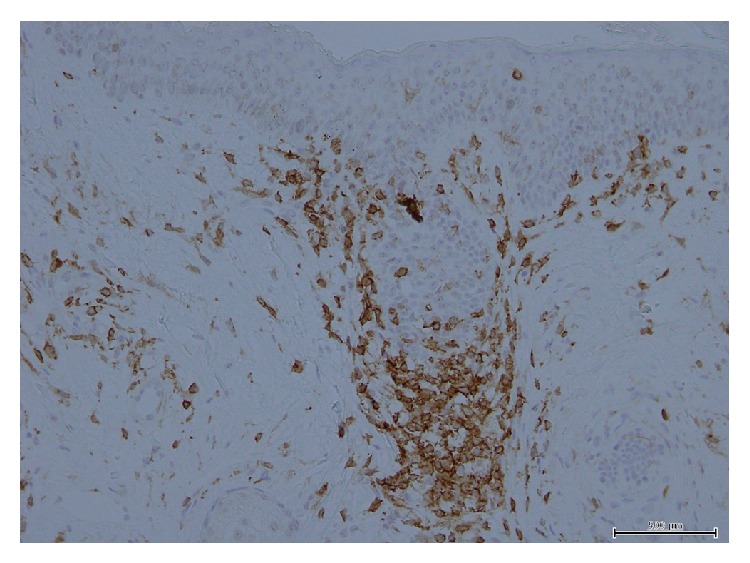
Microscopic findings of skin biopsy; CD4+ cells were infiltrated. These cells were CD3+, but no CD56+ cells or EBER+ cells were detectable. Also, only a few cells in this dermal area were stained with TIA-1 and Granzyme B (data not shown).

We report here a 77-year-old Japanese man, who had been a carrier of HTLV-1 infection and treated for IDH with oral prednisolone (maximum dose was 40 mg/day) for nearly 10 years. When he was referred to us, he had fatigue, loss of appetite, and exacerbated facial eczema/edema as well as dermatitis at the neck/upper chest and nuchal area and upper back regions (Figure 1). He did not have any lymphadenopathy, hepatosplenomegaly, and clinical symptoms/signs of HAM/TSP. Laboratory data were as follows: WBC 4,700/*μ*L, Hb 12.3 g/dL, platelet counts 91,000/*μ*L, AST 74 U/L, ALT 45 U/L, LDH 882 U/L, BUN 16.5 mg/dL, creatinine 0.94 mg/dL, CRP 2.39 mg/dL, sIL-2R 1,280 U/mL, beta-2-microglobulin 6.5 mg/L, and ANA x40 positive, but complements were within normal. The patient had 22 copy/10*e*4 PBMCs of proviral HTLV-1 load (normal <20 copy/10*e*4), with positive anti-HTLV-1 antibody, consisting of western blot pattern of GP46 (+), p53 (+), p24 (+), and p19 (+). He showed a marked increase of granular lymphocytes with atypical nuclei in this peripheral blood (Figure 2) which were identified to be NK cells, with a phenotype of CD2+ (97%), CD3+ (17%), CD4+ (12%), CD7+ (94%), CD8+ (6%), CD19+ (0%), CD20+ (0%), CD11c+ (70%), CD16+ (82%), CD56+ (67%), HLA-DR+ (68%), and NKp46+ (36%). To rule out if these NK cells were in fact T cells with NK cell phenotype, we tested rearrangement of T-cell receptor (TCR) C*β*1 gene by southern blot analysis in the peripheral blood. Results showed no TCR rearrangement bands (data not shown). Thus, during the pretreatment period, absolute CD56+ NK cell counts in the peripheral blood were in a range of 986/*μ*L–1,270/*μ*L (reference values: 14–634/*μ*L). Bone marrow smear showed hypocellular and hypoplastic marrow, with decreased numbers of megakaryocytes; however no abnormal features were noted. Flow cytometry of bone marrow revealed NK cell dominance as seen in the peripheral blood. Analysis of his peripheral blood as well as bone marrow showed a normal karyotype. On the other hand, infiltrating mononuclear cells in his dermis obtained by the skin biopsy of facial eczema were shown to be CD3+CD4+CD56-EBNA-. Only a few cells in this dermal area were stained with TIA-1 and Granzyme B (Figure 3). These findings were compatible with smoldering and chronic ATLL skin lesions. The patient was given a combination of weekly etoposide (100 mg/body) with dexamethasone (4 mg/body) for a total of 5 courses, when his skin lesions were markedly improved. Thereafter, the treatment was continued up to a total of 12 courses. This treatment resolved skin lesions but did not affect his NK cell-dominant features in the peripheral blood (data not shown).

## 3. Discussion

The clinical and pathological features of HTLV-1-associated cutaneous disease are diverse [4]. Our patient had chronic dermatitis at the face, neck area, and the upper back over the period of ten years, in association with HTLV-1 antibody seropositivity but not with HAM/TSP as previously reported [5]. IDH is common in childhood; thus this case may not be a typical IDH, although report on adult-onset IDH was also available [6]. In patients with ATLL, peripheral blood generally shows the phenotype of CD2+CD3+CD4+CD7-CD8-, while in our patient it showed a pattern of CD2+CD3-CD4-CD7+CD8-CD16+CD56+, strongly suggesting NK cells. Although the possibility remained that these cells could be T cells with aberrant NK cell phenotype, lack of TCR C*β*1 rearrangement in these cells confirmed that they are actually NK cells.

In the past, Norris et al. demonstrated that in* in vitro* culture assay of 7 days, CD56+ NK cells spontaneously proliferated in response to HTLV-1. This spontaneous NK cell proliferation positively correlated with HTLV-1 proviral load, but not with the presence of HAM/TSP [7]. Besides this reactive NK cell proliferation, HTLV-1-infected NK cells were also documented; however, the HTLV-1-infected NK cells were not immortal and were phenotypically indistinguishable from their uninfected counterparts [8]. Coelho-dos-Reis et al. described a statistically significant increase, but not as magnificent as ours, of the macrophage-like subset (CD14 + CD16+) or NK cell subset in the HTLV-1-infected group with skin lesions. In their patients, high levels of proviral load were noted with an increase of NK cells [2]. By contrast, in our case, striking NK cell expansion was not associated with increased proviral load. The critical question in our case is whether these NK cells are reactive or neoplastic in nature. Ohshima et al. showed that HTLV-1-infected CD8+ and/or CD56+ cells probably confer no cytotoxic function [9]. Considering the fact that the NK cells in our case contain abundant granules, in association with barely detectable provirus load and normal karyotype, the expansion of NK cells in our case could be reactive and may play an immunoregulatory role preventing the progression of IDH to HAM/TSP. Treatment of IDH has not yet been established and there are still few treatment options [10]. In our case, we treated with a total of 12 courses of etoposide/dexamethasone. Although skin rash resolved significantly with this treatment, we hope that expanded NK cells may also act to maintain the confinement of the HTLV-1-positive CD4+ cells in the skin and prevent the progression from IDH to the HAM/TSP stage of the disease. However, since CD4+ T cells did not coexist with NK-cells in the skin biopsy sample, this beneficial effect may be achieved through cytokines released from expanded NK cells. Although a possibility may remain that NK cells could be infected with HTLV-1 in future, as demonstrated by Steve Lo et al. [8], it is assumed that our patient clinically verifies the previously observed* in vitro* phenomenon that HTLV-1 primarily drives expansion of CD56+ NK cells.
